# Epidemiology and Diagnostics of Cacao Swollen Shoot Disease in Ghana: Past Research Achievements and Knowledge Gaps to Guide Future Research

**DOI:** 10.3390/v16010043

**Published:** 2023-12-27

**Authors:** George A. Ameyaw, Owusu Domfeh, Ebenezer Gyamera

**Affiliations:** Cocoa Research Institute of Ghana (CRIG), New Akim-Tafo P.O. Box 8 E/R, Ghana; kofdomfeh@gmail.com (O.D.); eag42@cantab.ac.uk (E.G.)

**Keywords:** cacao swollen shoot virus, cacao, diagnostic, diversity, epidemiology, impact

## Abstract

Cacao swollen shoot disease (CSSD) caused by complexes of cacao swollen shoot badnaviruses (family *Caulimoviridae*, genus *Badnavirus*) remains highly prevalent and devastating in West Africa. The disease continues to impact substantially on cacao yield loss, cacao tree mortality, and decline in foreign exchange income from cacao bean sales. Currently, the disease is estimated to have a prevalence rate of over 30% in Ghana, as assessed in the ongoing third country-wide surveillance program. Although achievements from past research interventions have greatly elucidated the etiology, biology, epidemiology, diagnostics, and management of the disease, there are some outstanding knowledge gaps. The role of these information gaps and their effect on CSSD epidemiology and prevalence remain unanswered. This paper summarizes existing scientific knowledge from past research achievements that have provided elucidation on CSSD epidemiology, management options, and guided future research. The discussion highlights the need for multidisciplinary research with modern tools and institutional collaborators to holistically bring clarity on knowledge gaps on pathogen biology, virus–host-–vector interactions, role of environmental and soil nutrient effects on CSSD severity, evolution pattern, role of alternative hosts on virus species diversity, vector population dynamics, and their overall impact on CSSD prevalence and integrated management in cacao plantations.

## 1. Introduction

**Figure 1:**
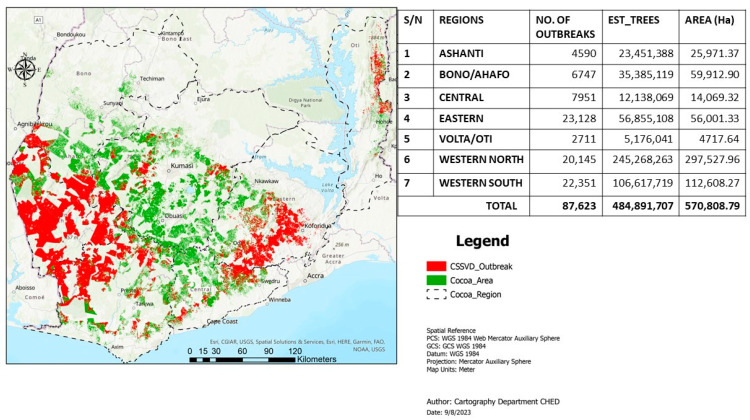
Current CSSD survey map in Ghana as documented by the Cocoa Health and Extension Division (CHED) of Ghana Cocoa Board in the ongoing third country-wide surveillance program. Courtesy, Cartography Unit of CHED, Survey Report, 2023.

Cacao swollen shoot disease (CSSD) remains a major cacao (*Theobroma cacao*, L.) production constraint in Ghana and Côte d’Ivoire and the other West African cacao-producing nations, including Togo and Nigeria. The disease, which is transmitted by several species of mealybug insects, could potentially reduce yield by approximately 30% to 50% and even cause death of cacao trees within 2 to 3 years of infection [1]. Although the virus was first identified in the eastern region of Ghana in 1936 [2], it is now widespread in all the major cacao-growing nations in West Africa. The disease continues to contribute to severe cacao tree mortality, most especially the western north and south regions where the bulk of Ghana’s cacao beans are produced [3]. Over the years, the disease has been managed through the rogueing technique, i.e., “cutting-out approach”, with the aim of removing scatter infections in the field [4]. This strategy aims at minimizing inoculum sources and disease pressure to ultimately decrease its spread to newly replanted cacao farms [4,5]. This control strategy has, however, persistently encountered numerous challenges emanating from farmer opposition, limited funding, and frequent discontinuities in the implementation of the program [5]. Cumulatively, these challenges have resulted in high CSSD prevalence and continuous spread to newly established cacao farms in Ghana, especially in the western regions, as documented by the Cocoa Health and Extension Division (CHED) of Ghana Cocoa Board in the ongoing third country-wide survey (CSSD survey map, CHED, 2023) (Figure 1).

Findings from past research interventions have significantly elucidated the etiology, epidemiology, diagnostics, and directed scientific research and recommendations on strategies for integrated management of the disease in West Africa. However, there are outstanding knowledge gaps on the contemporary biological processes and identity of the viral causal pathogen, symptomatology, and influence of environmental conditions on symptom severity. Also, issues of host–pathogen–vector dynamics, virus species diversity, geographical distribution, and their interactive impact on cacao production have not been adequately clarified from past research. These existing information gaps hamper the deployment of effective CSSD management strategies aimed at minimizing the effect of the virus on cacao production in Ghana and the other producing nations in West Africa. This paper thus provides a review of existing research knowledge that has over the years clarified the status of CSSD etiology, epidemiology, serological, and molecular diagnostics of the disease. Prominent knowledge gaps and their effect on CSSD management are however highlighted and discussed. The paper emphasizes the need for further scientific investigations on the prevailing information gaps for better clarity on their impact on CSSD prevalence, symptom severity, and integrated management in the field.

## 2. CSSD Etiology and Physicochemical Features

The earliest scientific breakthrough on CSSD research at the Cocoa Research Institute of Ghana (CRIG) was the identification and confirmation of the then putative “swollen shoot” symptoms reported in farmers’ farms to be caused by a viral pathogen [2]. The causal pathogen was subsequently named the “cacao swollen shoot virus” (CSSV) in 1940 [6]. The physicochemical properties of the virus as determined from the earliest purified preparations indicated that the virus particles are organized in a non-enveloped bacilliform fashion, with sizes ranging between 121 × 28 nm and 128 × 28 nm. Other properties of the virus comprised a sedimentation coefficient of 218 S20w; thermal inactivation point (TIP) between 55–60 °C; and longevity in vitro (LIV) of 28–85 days (at 2 °C) [7,8]. The virus was reported to be inactive in causing infection at 50 °C after 10 min of continuous exposure to this temperature [8]. Infectivity of virus preparations was sustained after 24 h storage and was retained after 96 h at 0–4 °C, but was lost at 25 °C and at 1/100 dilution. Virus infective material (viral particles) was also precipitated by half saturation with ammonium sulphate at 25 °C and determined to be stable between pH 6.0 and 8.0 [7].

Further improvements in efficiency associated with CSSV purification protocols enabled the development of the first antisera for the virus for utilization in enzyme-linked immunosorbent assay (ELISA), immunosorbent electron microscopy (ISEM), and virobacterial agglutination tests (VBA) tests as protein-based diagnostic techniques for the virus [9,10]. Availability of purified viral particles led to the commencement of mechanical inoculation techniques for laboratory transmission of the virus for purposes of assaying for evidence of infections with suspected samples [8,9,10]. The efficiency of the mechanical transmission system was subsequently enhanced, and it was used as a tool to screen cacao germplasm materials in resistance breeding activities by the CRIG [11]. Based on these detection techniques, different types of the virus (mainly called strains/isolates) are recognized and characterized to exist in the different cacao growing regions of Ghana and the other growing nations in West Africa [9]. Accordingly, names of CSSV strains/isolates, as kept in the ‘CSSV museum’ of the Cocoa Research Institute of Ghana (CRIG), are labeled based on geographic location of first collection and symptom severity [8,10].

## 3. Genome Organization of CSSV

**Figure 2:**
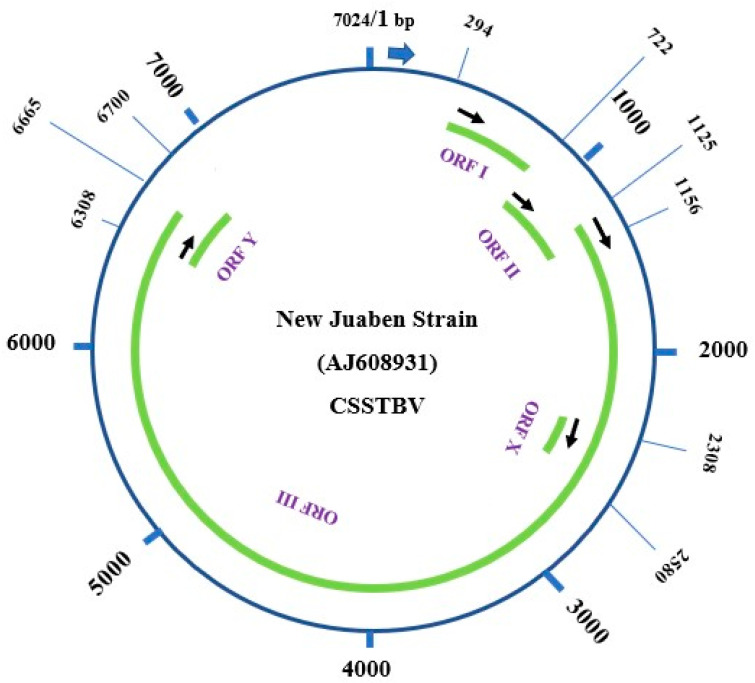
A schematic representation of the full-length genomic organization of the New Juabeng strain of the virus depicting positions of the various ORFs, i.e., 1, 2, 3, X, and Y, and the estimated size of the whole genome. This was generated with the latest New Juabeng sequences as deposited in the GenBank with Accession no. AJ608931.

Earlier molecular investigations confirmed CSSV to be a double-stranded DNA virus, and it was taxonomically classified to be a member of plant virus family *Caulimoviridae* and genus *Badnavirus*, with double-stranded circular DNA (dsDNA) [12,13,14]. The complete genome sequence of the various strains and isolates of the virus, including the most characterized New Juaben Strain from Ghana, indicated that the genome has been organized into five main putative open reading frames (ORFs 1, 2, 3, X, and Y) on the plus strand of the genome [13,14,15] (Figure 2). Individually, the ORFs code for specific proteins with or without recognized functions [14,15]. For instance, ORF1 encodes a 16.7 kDa protein, whose function has not been clarified. ORF2 encodes a 14.4 kDa nucleic acid- binding protein [15]. ORF3 also codes for a 211 kDa polyprotein which contained several products such as an RNA binding domain, consensus sequences for cell-to-cell movement proteins, a reverse transcriptase (RTase), an aspartyl proteinase, and an RNase H [13,14,15]. The latter two ORFs, X (13 kDa) and Y (14 kDa), were, respectively, noted to overlay ORF3 and encode proteins of unfamiliar functions [12,13,14,16]. The genome size of the various isolates/strains of the virus from field infections generally varies from 7024 bp for isolate N1A to 7242 bp, with the ORF1 being the most conserved coding region compared to the other coding regions [13,14]. Notably, the first region of ORF3, which encodes for movement proteins, is highly conserved, comparable to other para-retroviruses, while the other regions of ORF3 had an intermediate level of variability [13]. Availability of information about the features of the different genomic regions of the virus and their variability provided the foundation for the development of specific and generic polymerase chain reaction (PCR) primers for the detection of the virus [13]. Nonetheless, further updates on genomic information about the more conserved regions of the ORFs of the virus are urgently required. This is based on the availability of more than 80 full-length genomic sequences currently deposited in the GenBank from over eight different species of the virus, in contrast to the few full-length sequences previously considered in the literature on this subject [13].

## 4. Characterization of Novel CSSD Badnavirus Species

Availability of modern sequencing techniques, including the utilization of next-generation sequencing (NGS) technologies coupled with advanced bioinformatics analyses, have resulted in enhanced knowledge and better understanding of the full-length genomic information of the existing cacao badnavirus in field infections [17]. Consequently, this has led to the identification and further cataloguing of over eight cacao badnavirus species to be associated with cacao plants in West Africa [17,18,19]. The key cacao badnavirus species reported to be prevalent in current field infections in Ghana and Côte d’Ivoire include *Cacao swollen shoot Togo A virus* (Cacao swollen shoot Togo A virus; CSSTAV), *Cacao swollen shoot Togo B virus* (Cacao swollen shoot Togo B virus; CSSTBV, *Cacao swollen shoot CD virus* (Cacao swollen CD virus; CSSCDV), *Cacao swollen shoot CE virus* (Cacao swollen shoot CE virus; CSSCEV); and *Cacao swollen shoot Ghana M virus* (Cacao swollen shoot Ghana M virus; CSSGMV) [17,19,20]. The evolutionary pattern associated with these different cacao badnavirus species and their relationship with the previously known species of the virus remains to be validated [17]. Development of PCR methodologies for these different cacao badnavirus species has progressed steadily over the years to become the preferred diagnostic tool for the virus [17,21]. Nonetheless, the detection potential of the available PCR primers has been erratic, with varying detection efficiencies across field samples [22]. Ultimately, further improvement in the efficiency of these PCR diagnostic tools is critically needed to complement screening of cacao germplasm for breeding of tolerant planting materials for farmers and to support the implementation of field management strategies for the disease. Thus, unremitting sequencing of samples from field infections would inevitably result in better identification and characterization of different species and strains of the virus involved in current infections for improvement and optimization of molecular protocols for high detection efficiencies [17]. This information will help in further elucidation of knowledge gaps on the pattern of distribution of the known cacao badnaviruses relating to the presence of alternative wild host types, vector population dynamics, and the impact of prevailing environmental factors, which would be well understood through sustained research. Consequently, application of HTS with advanced bioinformatics techniques is critically needed to unravel the complex biological processes linking CSSD etiology and epidemiological dynamics across the different geographical cacao landscapes.

## 5. CSSD Symptomatology and Mixed Infections in the Field

Different kinds of visible symptoms are expressed in CSSD-affected cacao plants and this is mostly dependent on several factors, including virus species and strains involved in infection, host genotype, and environmental features [23,24]. Contingent on these factors, the types of visible symptoms expressed in affected cacao plants vary in severity and might include the characteristic stem swellings, root swellings, and pod deformation, with or without leaf symptoms [24]. The commonest leaf symptoms include red vein banding of the immature “flush” leaves, followed by chlorotic vein flecking or banding which may occur in angular flecks [24]. Stem swellings could occur at the nodes, internodes, or tips of shoots, and some severe strains sometimes cause infected pods to change shape and become rounder, smaller, and with a smoother surface [23,24]. Infections from less severe (mild) isolates of the virus, however, produce only transient leaf symptoms, and sometimes induce marginal stem swellings on affected cacao plants with little effect on yield [25,26]. The occurrence of mixed and co-infections with different species and strains sometimes makes it difficult to attribute visible symptoms to specific species or strains of the virus in field infections. Delineating the impact of individual viruses and/or synergistic effects on the severity of symptom expressions from mixed and co-infections on growth and yields of CSSD-affected cacao plants has not been well studied. Moreover, the possible beneficial role of mixed co-infections in conferring immunity and symptom suppression is poorly understood.

Visual examination of affected or suspected cacao plants and other hosts for evidence of visible leaf and stem symptoms has been the foremost method for field and graft-based recognition of suspected infections in field samples of CSSV disease [27]. This practice involves the constant lookout for identifiable symptoms of CSSV to confirm or reject infection status of suspected diseased plants. Visual inspection is considered ineffective, as latently infected hosts, i.e., infected but not expressing observable symptoms, are sometimes missed, and the suspected cacao plants are counted as non-infected. It is also known that successful detection of an infection with visual inspection might be subjective and may be reliant on the level of expertise of the inspector, as well as the physiological condition of the suspected host at the time of inspection [27]. Furthermore, results from visual inspection could be unreliable as they could be prejudiced by nutritional deficiencies and other physiological factors of the affected host plants which may cause somewhat similar leaf symptoms analogous to virus infection. Constant observation for appearance of visible symptoms is also time-consuming, as suspected disease plants sometimes to need to be grafted onto healthy counterparts (Amelonado cacao cultivars) and frequently monitored and assessed for a period of not less than 3 years before an infective status can be confirmed or rejected.

Consequently, there is the need for a quicker and more reliable detection system for the virus, and the lack of such efficient diagnostics techniques has been a major limitation with regards to CSSV epidemiology and management. Currently, there is a drift towards molecular diagnostic tools, as the efficiency, repeatability, sensitivity, and reliability associated with molecular detection methods are constantly being improved with modern techniques and protocols which tend to lower the detection time. Molecular detection procedures provide faster results which accordingly enables more field samples to be assessed within a shorter period for prompt decision making without necessarily depending on visible symptom expression. Presently, there is a lack of clarity on the interaction among biological processes of the key cacao badnavirus species, the occurrence of mixed infections, and the influence of environmental parameters on yield loss of affected cacao plants. Similarly, there is limited information on the impact of cacao virus mixed infection on virus titer loads and symptom expression. Ultimately, there is the need for advanced scientific investigations for better elucidation of the epidemiological importance of the highlighted gaps in knowledge on CSSD symptom severity and yield decline.

## 6. Mealybug Insect Vectors of CSSD Badnavirus and Control Challenges

Over 14 species of mealybug insects (Pseudococcidae, Homoptera) have been identified as plausible vectors for the virus and thus responsible for semi-persistent transmission by feeding on all parts of infected cacao trees, including flowers, cherelles, pods, and leaves [28,29,30]. The immature adults of the mealybug (commonly referred to as nymphs or crawlers) are able to transmit the virus [29]. The mealybugs obtain the virus inoculum from infected host tissues during feeding, and subsequently cause infection in healthy cacao trees through transmission feeding [30]. However, mealybug transmission success and efficiency are limited by many factors, such as insect feeding preference, available mealybug species, mealybug age, available viral titer, and host morphological features [30]. An estimated acquisition-access feeding of at least 16 to 24 h is needed for mealybugs to become viruliferous [29,30]. Once the mealybugs acquire the virus, it can be transmitted to a susceptible host within few hours, and they could remain viruliferous for about four days post-acquisition [30]. The mealybug transmission assays require constant maintenance of mealybug colonies in the laboratory through frequent field collections, are labor-intensive, and can yield variable results due to variances in virus-vector transmission proficiencies. The two most efficient mealybug transmitters of the virus, i.e., *Formicococcus njalensis* and *Planococcus citri*, predominate and constitute about 90% of all mealybug populations within the cacao landscape in Ghana [31]. Nonetheless, the influence of the population dynamics of the mealybugs, in terms of feeding preference on the cacao hosts relative to environmental differences, on CSSD prevalence and symptom severity have not been well clarified and thus remain understudied.

Initial efforts to control the mealybug vectors with orthodox insecticides achieved partial success with nicotine, parathion, or DDT-based insecticides [32,33], albeit this was accompanied with problem of cacao bean tainting with the chemicals, causing reduced quality and marketability due to high residue levels in the cacao beans. The use of these classes of chemicals on the mealybugs was therefore discontinued. Further investigations of other chemicals, including Aldrin, Dieldrin, and Chlordane, indicated that these chemicals were very toxic to the mealybug attendant ants on cacao. Although application of these chemicals led to a reduction in the number of ant-attended mealybug colonies, regrettably, there were underlying side effects on other insect pests, with an upsurge in the population of pod borers and leaf miners [32]. These multifaceted factors limited the possibility of controlling the mealybug vectors using contact insecticides. Correspondingly, early efforts at biological control were ineffective [34,35]. The use of the fungal strain *Aspergillus parasiticus* (Speare), which had been reported with the potential to kill the mealybug vector *P. njalensis* in the laboratory, had little success in subsequent field experiments. Predatory insects, including *Anagyrus kivuensis* from Kenya and six other hymenopterous parasites, were introduced into Ghana, bred in large quantities, and released into farms. Unfortunately, only one of the species, *Pseudoaphycus angelicus* (Howard), established permanently, and it did not considerably decrease mealybug populations [36]. Further attempts to control the mealybugs with the use of parasitoids and local predators have had little success in Ghana or elsewhere [36]. Effective management of the mealybug vector remains critical in the integrated management efforts of the virus and thus requires further investigations with modern technology.

## 7. CSSD Alternative Host Plants Research

Evidence from earlier research activities suggested that CSSV might have originated from wild forest and indigenous trees within the cacao ecosystem [37]. It was considered that the virus might have crossed over into the cacao plant when it was first introduced into the various growing regions [37,38]. These assertions were based on earlier investigations on alternative host plants in the Western Region of Ghana which suggested that CSSV infections into cacao plants possibly originated from *Cola chlamydantha* (K. Schum) trees [37]. It was reported that *C. chlamydantha* trees were found infected with CSSV, both within cacao farms and miles away in forest reserves [37,38]. These wild (forest tree) hosts were thus assumed to have served as potential sources (reservoirs) of inoculum of the virus to the prevailing and wandering mealybug insect vectors, and were probably the original hosts of this virus before cacao was introduced into West Africa [37]. Subsequently, other tree species that were identified as plausible wild alternative hosts of the virus included *Erythropsis barteri* (Mast), *Sterculia trangacantha* (Lindle), *Sterculia rhinopetala* (K. Schum), *Cola gigantea* var. *glabrescens*, (Bronnan et Keay), *Adansonia digitata* (L.), *Bombax buonopozense*, and *Ceiba pentandra* (L.). The most common wild hosts in the cacao plantations in Ghana included *C. pentandra* (L.), *A. digitata*, and *C. gigantea* [38]. These were considered the most significant natural sources of infection, as these wild tree species occurred predominantly in all the cacao growing areas of Ghana at the time of these investigations [38]. It is, however, noteworthy to mention that not all the suspected wild hosts could be considered good sources of virus. Accessibility of the virus inoculum to the mealybug vectors declines to a low level in these alternative hosts and might not be readily available to mealybugs. Coppicing (cutting off the tops of the trees near the base of the crown) of the matured alternative host plants resulted in a temporary upsurge in virus concentration and availability to the mealybug vectors for transmission from the wild hosts to cacao plants and vice versa [38].

Based on the initial evidence of the potential involvement of the wild (alternative) host plants in CSSV spread, it was therefore recommended that the implicated wild host plants should thus be promptly removed as much as possible from cacao plantations [37,38]. This recommendation has not always been fully adopted by the farmers and stakeholders involved in CSSD management programs, and is thus considered as one of the main challenges that accounts for the continuous spread of the virus in the field [37,38]. Updated information on the status of the many forest trees species, shrubs, and weed species in the cacao ecosystem as plausible alternative hosts or otherwise remain critical for the continuous implementation of a sustainable integrated management strategy for the disease.

## 8. Germplasm Screening for Tolerant/Resistant Planting Materials

Availability of resistant/tolerant cacao varieties for establishment of new cacao plantations has long been suggested as the most feasible strategy and surest means to minimize CSSV spread in the field [39]. This strategy is anticipated to reduce the impact of the disease and the damage it causes to affected cacao plants as a long-term management for the virus [39]. Based on this assertion, series of germplasm screening tests have been carried out, involving local cacao cultivars and others introduced from a germplasm collection maintained in Trinidad. Thus far, none of the tested cacao varieties have been reported to be immune or resistant to the virus. It is, however, known that hybrids of Upper Amazon parentage were more difficult to infect with the virus than the local Amelonado variety [40]. Even when infected, the Upper Amazon hybrids developed only mild symptoms, thus indicating some degree of tolerance to infection [40,41].

The Upper Amazon varieties were therefore introduced in the early 1950s and multiplied for farmers to be used in new plantings to substitute for the Amelonado variety, which hitherto had been the foremost cultivars grown by the farmers [39]. These varieties were later found to have low tolerance to CSSV [41]. The F3 Upper Amazon and the later series II hybrids (Upper Amazon X x Amelonado and Upper Amazon X x Trinitario) were to a lesser extent susceptible than the Amelonado variety. These hybrids performed well against the hostile effects of the virus, even though none of the parents was specifically selected for resistance against CSSV. Further greenhouse and field trials were performed in the search for tolerant or resistant progenies in Ghana and Nigeria, respectively [42,43]. It was reported in these studies that the Parinari genotype and its hybrids showed a characteristic necrotic leaf symptom in Nigeria, which was considered not to be a true hypersensitivity, while Amelonado, Morona, and Trinitario clones were noted to be highly susceptible to the virus [44,45]. The Upper Amazon and Scavina clones were less susceptible than Nanay and Iquitos types, which were only slightly susceptible. It was found in further studies in Ghana that progeny of Trinidad pod T17 of Amazon parentage (Iquitos) might be resistant to the virus, as previously suggested in some earlier studies [46,47].

Thus, resistance breeding programs became the main research focus of the British Research Team (BRT) sent to Ghana between 1969 and 1978 to help reverse the CSSV-induced decline of the cacao industry with resistant and tolerant cacao varieties of the virus [43]. Through their sustained research efforts, some hybrids of Inter-Upper Amazon parents involving their male parents were selected in 1978 for resistance to CSSV, even though selection of female parents was constrained by the need to use existing seed gardens [47,48]. These Inter-Upper Amazon hybrids were generally more resistant to CSSV infection than the equivalent series II hybrids, and were therefore recommended for farmers to use in new cacao plantings [48]. Subsequent studies suggested that the level of resistance of these varieties to CSSV was not adequate for long term protection from infection of the virus [49]. The search for cacao varieties with improved resistance to the virus, through screening of existing and new cacao germplasm, mutation breeding, tissue culture techniques, and other modern breeding tools, has been a constant research priority at CRIG [49,50,51]. This is aimed at developing high performing varieties which can withstand the prevalence of high virus inoculum to complement efforts at integrated management of the virus in the field [50,51,52].

## 9. Mild Strain cross Protection Research

Observations from earlier research activities indicated that young cacao trees found around outbreaks of virulent virus isolates (New Juabeng strain) could be protected with mild forms of CSSV [53,54,55]. It was thus suggested that a mild strain protection method could be applicable in areas where the disease was highly prevalent and spreading uncontrollably [53]. Nonetheless, the main concern at that time was that mild virus might mutate after a period to cause serious devastation [54]. Mild strain protection approach was therefore considered not to be compatible with the intention to treat all outbreaks by cutting out sources of infection [56]. It was thus suggested that comprehensive research need to be carried out on the behavior and dissemination of the mild strains and their long-term protective effect on cacao [56].

Nonetheless, further research on mild strain research at CRIG has not progressed as expected, due to limited information and other concerns about the available mild strain-inoculated cacao trees being able to harbor severe strains at the same time [55], and also the probability of mild strains being mutated to change from less severe to severe strains in the field [57], as well as the fear of mild strain protection being temporary, and the challenges in differentiating between field symptoms induced by mild and severe strains to warrant prompt eradication of severe field infections [56]. These concerns and challenges were supposed to be investigated through long-term on-station trials at CRIG before a conclusive recommendation could be made for large-scale application by farmers [57]. Available information from the on-station investigations indicates that the apparent protection by the two main mild strains (i.e., N1 and SS365B) in conferring immunity to cacao plants breaks down between 15 years and 20 years post inoculation [58,59]. This finding suggests the need for repeated mild strain inoculations for the sustainable and long-term protection of cacao plants in the field [57,58,59]. Issues about the need for the development of inoculation approaches that would ensure efficient introduction of the mild virus into cacao seedlings prior to distribution and planting were highlighted in these reports. Collectively, this updated research information supports the need for better clarity of the status of the mild strain phenomenon and its associated challenges before its adoption as a management strategy for the virus in the field.

## 10. Molecular and Serological Diagnostics of CSSD Badnaviruses

Approaches for efficient detection and diagnoses of viruses are important requirements for virological investigations and development of satisfactory control measures [57]. Determinants of effective viral diagnoses and detection include evaluating prevalence within a locality and recognizing new virus strains and species [57]. Some of the common techniques utilized in the past for field and laboratory screening for CSSD include visual inspection for symptoms, graft indexing, vector transmission tests, and different serological/molecular detection tests [60,61]. The principal serological technique commonly used for laboratory screening for CSSD is the enzyme-linked immunosorbent assay (ELISA). This involves the use of polyclonal antisera raised in rabbits from CSSV purified from several of the strains and isolates presently available [61]. However, the problem of low virus titer in CSSV purifications from infected tissues most often plays a role in making several antisera unspecific for screening and detection of the virus [61]. This sometimes results in issues of false negatives (virus concentration too low) and false positives (background reaction too high) from ELISA serological tests. This is because the antisera may contain antibodies raised against the host plant material rather than the virus, thus leading to high background reactions. Furthermore, use of ELISA for CSSV detection in healthy but suspected plants having mild strain infection or purifications with low virus titer value have been ineffective over the years [10]. The use of virobacterial agglutination (VBA) as a CSSV diagnostic technique gained prominence in the 1990s due to its apparent sensitivity compared to the ELISA technique [9,10]. VBA testing involves the use of protein A on the surface of the bacterium *Staphylococcus aureus* which has a particular affinity for part of the Y-globulin which is part of the antibody molecule. Although VBA is easily applied as a detection tool for CSSV, the issue of high background reactions resulting from non-CSSV antibodies makes detection of some CSSV strains very difficult [62]. At present, the drift is toward the use of various forms of polymerase chain reaction (PCR) methodologies for disease diagnosis, due to recent advances in nucleic acid-based virus detection tools which are considered highly sensitive, repeatable, and reproducible.

Over the years, further improvements in PCR methodologies have significantly resulted in the development of cost-effective tools for the diagnosis of CSSD badnaviruses [17,18,20,21]. The efficiency and sensitivity of the available detection methods, however, remains particularly challenging, due to apparent species variability, low virus titer, and high molecular diversity associated with most of the known cacao badnaviruses [25]. Further improvement of the PCR methodologies is greatly dependent on better characterization of CSSD species identity through advanced sequencing and polymerase chain reaction (PCR) methodologies [17,22]. Available advanced techniques that could be adopted for CSSD detection include immunocapture-PCR (IC-PCR), which combines the specificity of antibodies for trapping viral particles with increased sensitivity of nucleic acid (DNA) sequences [63,64,65].

Utilization of rolling circle amplification (RCA) assay as a genomic DNA pre-enrichment technique is a known strategy to circumvent problems of polysaccharides and phytophenolic inhibitors that are usually co-extracted with nucleic acids. RCA techniques could thus be adopted for enhanced PCR detection of CSSD, as has been reported for other plant viruses [65]. The high efficiency associated with the loop-mediated isothermal amplification (LAMP) assay as a molecular detection tool compared to the conventional PCR assay is well documented [66,67]. The LAMP and HTS techniques could thus be considered and explored for enhanced CSSD diagnostics and further detection of endogenous viral elements (EVEs) in the cacao genome [21]. The use of these modern techniques is anticipated to augment rapid germplasm screening and early detection during field eradication activities in the rehabilitation of CSSD-devastated farms.

## 11. Environmental and Edaphic Factors on CSSD Epidemiology

Soil nutrition is considered one of the manageable factors of the environment that have been postulated to influence plant resistance or tolerance to pathogenic attack [68]. Nonetheless, the possible effects of soil nutrients on CSSD symptom expressions have not been adequately clarified, with several unanswered questions about the role of soil nutrition on CSSD epidemiology [69]. Similarly, there is inadequate evidence on the role of environmental parameters, such as level of shade, and their interactive effect with soil nutrient status on CSSD epidemiology. Although the apparent positive effect of adequate level of shade (i.e., 30 to 50% light penetration) in reducing severity of CSSD symptoms was previously reported [59,70], limited studies have subsequently been carried out on this subject. Thus far, the combined effect of soil nutrient status and shade level on CSSD remains poorly understood. It is therefore imperative that a controlled field experiment is initiated to understand the interactive effect of soil nutrient status and environmental factors on CSSD-affected cacao plants across different agroecological zones in Ghana and the other West African cacao-producing nations.

## 12. Discussion

This paper reviewed and outlined past and current research interventions that have elucidated the identity of cacao badnaviruses involved in field infections and their impact on CSSD prevalence, symptom severity, and cacao production in West Africa. Emphasis was placed on the prevailing knowledge gaps associated with the biological processes of the known virus species, their diagnosis, effect on disease progress, and prevalence. These information gaps emanate from limited scientific research in these thematic areas, due to inadequate funding and limited laboratory tools as submitted by [66]. The paper thus proposes sustained research interventions to investigate the existing knowledge gaps to guide decisions on effective field diagnostics and management of the disease [23]. Strategically, these investigations need to target key research areas for clarity on the interaction between virus identity, host genotype–virus interactions, and the effects of environmental parameters on disease progress. Similarly, the role of alternative hosts, vector population dynamics, and their impact on CSSD spread and symptom severity require further investigation. Optimization of molecular diagnostic techniques for efficient, reliable, and repeatable detection of the known cacao badnavirus species and their evolutionary pattern also require urgent research attention [22].

Thus far, the key CSSD badnavirus species identified to be predominating in field infections in Ghana and Côte d’Ivoire include *Cacao swollen shoot Togo A virus* (Cacao swollen shoot Togo A virus; CSSTAV), *Cacao swollen shoot Togo B virus* (Cacao swollen shoot Togo B virus; CSSTBV), *Cacao swollen shoot CD virus* (Cacao swollen CD virus; CSSCDV), *Cacao swollen shoot CE virus* (Cacao swollen shoot CE virus; CSSCEV), and *Cacao swollen shoot Ghana M virus* (Cacao swollen shoot Ghana M virus; CSSGMV) [18,19,20]. As highlighted in this paper, the evolutionary pattern and epidemiological dynamics associated with these dominant cacao badnavirus species relative to the occurrence of alternative hosts in the cacao landscape remains poorly understood. It is hypothesized that many of these species might have spread from historical exchange of infected planting material and vector-infested cacao pods on farms, albeit this has not been confirmed through systematic research [18]. This assertion therefore requires further scientific investigations and confirmation with modern molecular techniques and enhanced bioinformatic tools. Better clarity on the identity of CSSD badnavirus species/strains, mild strains, and their evolutionary pattern relative to geographical distribution, species diversity, and mixed co-infections would ultimately complement molecular diagnostics and field management of the disease [18].

Investigations on vector transmission efficiency have been complex and laborious to perform and time-consuming, hence the resultant wide gap in knowledge on the various dynamics of mealybug transmission of CSSD badnaviruses in the cacao ecosystem. The interactive role of mealybug population dynamics and host factors for efficient transmission in the field have not been well clarified and thus need to be further investigated. There is limited information on the role of endogenous viral elements (EVEs) [21] on CSSD epidemiology, and this requires further investigations through a combination of the various –*omics* techniques and field experimentation. Availability of research information will elucidate the infectious status and pathogenicity of the identified cacao EVEs on vertical transmission of CSSD, as documented in other field crops such as bananas [66,67,71]. Clarifying the pathogenic status of cacao EVEs is essential to guide future research and quarantine decisions on cacao planting material (seedling and pod) movement, breeding programs, and certification of cacao seedlings for field planting.

Understanding the role of soil nutrient status and their interactive effect with other environmental parameters such as level of shade on the epidemiology of the cacao swollen shoot virus disease (CSSD) remains a research priority across the different agroecological zones. Although past studies have reported the positive effect of adequate level of shade in reducing CSSD symptom severity in Ghana [70], there is limited information on the combined effect of soil nutrient status and shade level on CSSD epidemiology in the medium to long term. Similarly, the influence of farm management and cultural practices on CSSD progress has not been adequately clarified from past research. Consequently, further research interventions are thus advocated on the influence of soil nutrition and shade level on CSSD incidence and symptom progress in mixed cocoa hybrids for better clarity on this subject. This will invariably complement the integrated management strategies for the virus for sustainable cacao production across the different agroclimatic zones in West Africa. Furthermore, application of modern technologies such as artificial intelligence, robotics technology, big dataset algorithms, image acquisition technologies, and remote sensing tools is advocated for CSSD surveillance, early detection, and prompt management [72].

## 13. Conclusions

This paper advocates for better clarity on the role of molecular properties of the existing cacao virus species, their geographical distribution, their prevalence, the influence of environmental (abiotic) factors, and their overall impact on CSSD epidemiology and diagnostics in the field. To this end, comprehensive CSSD badnavirus characterization and diversity research with modern tools aimed at gaining better understanding of the identity of key virus species, vector population dynamics, alternative hosts, and cacao genotypes predominating in current field infections is necessary. Further investigations on effects of environmental and soil nutrient factors and their role on occurrence of differential outbreaks across the different geographic regions of Ghana and Côte d’Ivoire remain a research priority. Thus, a multidisciplinary research strategy on the virus and the vectors involving multinational collaborators is hereby proposed to address the prevailing knowledge gaps and challenges in CSSD diagnostics and field management for sustainable cacao production.

## Data Availability

The data presented in this study are available upon reasonable request from the corresponding author.
